# Portable bacterial identification system based on elastic light scatter patterns

**DOI:** 10.1186/1754-1611-6-12

**Published:** 2012-08-28

**Authors:** Euiwon Bae, Dawei Ying, Donald Kramer, Valery Patsekin, Bartek Rajwa, Cheryl Holdman, Jennifer Sturgis, V Jo Davisson, J Paul Robinson

**Affiliations:** 1 School of Mechanical Engineering, Purdue University, West Lafayette, IN, 47907, USA; 2 School of Electrical and Computer Engineering, Purdue University, West Lafayette, IN, 47907, USA; 3 Visiting researcher, Cytometry Laboratory, Purdue University, West Lafayette, IN, 47907, USA; 4 Dr. J. Paul Robinson Purdue University Cytometry Laboratory, Bindley Bioscience Center, Purdue University, 1203 West State Street, Discovery Park, West Lafayette, IN, 47907, USA; 5 Department of Basic Medical Sciences, Purdue University, West Lafayette, IN, 47907, USA; 6 Department of Medicinal Chemistry and Molecular Pharmacology, Purdue University, West Lafayette, IN, 47907, USA; 7 Weldon School of Biomedical Engineering, Purdue University, West Lafayette, IN, 47907, USA

**Keywords:** Portable bacterial identification, Forward scattering, Colony count, Automation

## Abstract

**Background:**

Conventional diagnosis and identification of bacteria requires shipment of samples to a laboratory for genetic and biochemical analysis. This process can take days and imposes significant delay to action in situations where timely intervention can save lives and reduce associated costs. To enable faster response to an outbreak, a low-cost, small-footprint, portable microbial-identification instrument using forward scatterometry has been developed.

**Results:**

This device, weighing 9 lb and measuring 12 × 6 × 10.5 in., utilizes elastic light scatter (ELS) patterns to accurately capture bacterial colony characteristics and delivers the classification results via wireless access. The overall system consists of two CCD cameras, one rotational and one translational stage, and a 635-nm laser diode. Various software algorithms such as Hough transform, 2-D geometric moments, and the traveling salesman problem (TSP) have been implemented to provide colony count and circularity, centering process, and minimized travel time among colonies.

**Conclusions:**

Experiments were conducted with four bacteria genera using pure and mixed plate and as proof of principle a field test was conducted in four different locations where the average classification rate ranged between 95 and 100%.

## Background

Microbial identification is essential in various bioscience-related areas such as biosecurity, food safety, and monitoring and prevention of nosocomial infections. In general, three steps are needed to deliver correct identification of a species: sample acquisition/preparation, microbe detection, and microbe identification. Throughout the years, most effort in instrument development using optical technology has been focused on the development of a colony counter, which is essentially a detection device; further testing is required for identification. To identify and classify bacterial colonies, morphological methods, to observe characteristics of the bacterial colony *via* visual inspection, are widely studied and applied [1-3]. Previous studies employed a number of image-processing tools [4-8] to realize the optical colony counting process, including distance transforms, adaptive thresholds, modified Hough transforms, etc. In recent years, prototype systems and software for simple and rapid automatic counting of bacterial colonies have also been developed. Liu et al. [9] adapted an imaging system originally developed for ELISPOT assays to count colonies automatically and used their system to measure serum bactericidal activity (SBA) in human sera. Later, Putman et al. [10] showed that automated colony counters can process images of plated bacterial colonies obtained with digital cameras or document scanners, so that small laboratories without a colony counter can send an image of a plate via the Internet and receive an accurate colony count from a remote laboratory that own a dedicated counting software. Recently, Clarke et al. [11] presented a counting system equipped with a software called NICE (NIST’s Integrated Colony Enumerator), which combines extended minima and threshold algorithms to locate colonies and distinguishes touching colonies. Chen and Zhang [12] proposed an automatic bacterial colony counter that can detect dish/plate regions, identify colonies, separate aggregated colonies, and report colony counts. In their paper, they used a typical morphology-based system, a one-class support vector machine (SVM) with radial basis function (RBF) as the colony classifier. However, in the proposed instrument, the incident laser beam is capable of extracting the microscopic morphological differences between bacterial colonies that are visually indistinguishable.

Most previous research focused only on counting the bacterial colony and consequently required further steps to identify the actual bacterial species. Our proposed instrument not only counts the colonies but also captures forward-scatter patterns to identify them; this is the crucial advantage over those systems already mentioned. The reported system embodies the design concept which employs Elastic Light Scatter (ESL) patterns for bacterial identification. Early work by Guo [13] and Banada et al. [14] inspired the development of a forward scatterometer–based detection and identification technology. The biophysical understanding of the elastic light scattering was reported by treating the individual bacterial colonies as biological spatial light modulators [15-17]. Briefly, the incoming wavefront of the light source interacts with the microscopic features of the bacterial colony and propagates to the imaging plane to provide a unique scatter pattern. No labeling agent is required to discriminate among different types of bacteria. The first reported forward scatterometer system required manual positioning of the incident beam and motion control to record the forward-scattering signatures, which is tedious, laborious, and inefficient. To increase the efficiency of the data acquisition process, Bae et al. [18] further developed a new version of the instruments that was fully automated.

In this report, we describe a portable forward scatterometer that is lighter in weight, is smaller in footprint, and provides wireless data transmission capability. These characteristics make the portable forward scatterometer a perfect instrument for field-deployable microbial identification as suggested by Robinson et al. [19]. To simulate this situation, a field test was conducted where ELS patterns captured remotely were electronically transmitted for classification and identification. Compared to the previous instrument, this system has a number of improvements. First, the instrument is designed with rotation-linear movements rather than two linear movements, resulting in lighter weight and smaller instrument footprint. Second, several image-processing schemes have been added or improved (Hough transforms and R-θ centering algorithm). Third, wireless communication capability enabling access to the scatter-pattern database was incorporated to enable detection and identification from remote locations.

## Results and discussions

### Hardware

**Figure 1:**
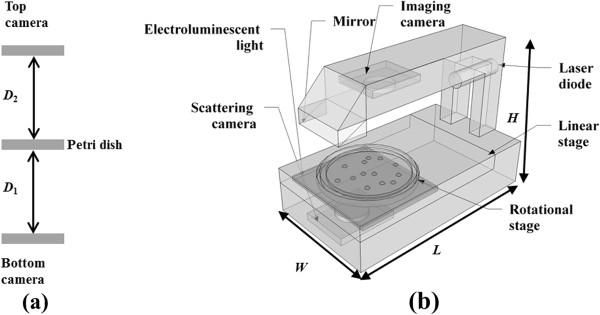
** Schematic diagram of the portable bacterial identification system using elastic light scatter patterns.** (**a**) The distance parameter D_1_(1.2 inch, from plate to the scatter camera) and D_2_ (4.5 inch, from imaging camera to the plate). (**b**) The system consists of a linear stage, a rotational stage, and two cameras (imaging and scattering), a laser diode of 635 nm, and electroluminescent light for back-illumination. Approximate size W × L × H = 6 in × 12 in × 10.5 in. The device weights about 9 lb.

The proposed system is 12 (L) × 6 (W) × 10.5 (H) inches and weighs 9 lb. It consists of three distinct parts: a monochrome camera located right above the petri dish holder and used for colony counting and locating, a forward scatterometer, and a motorized stage that can rotate and translate the petri dish in the *θ* and *R* directions. The top camera (PL-B741U, PixeLINK, Ottawa, Canada) has 1280 × 1024 resolution with a unit pixel size of 6.7 μm. The camera is connected to a PC (Pentium 4, CPU 2.80 GHz, 1.00 Gb of RAM) via a standard USB 2.0 interface. An image of the petri dish is sent to the custom built analysis software that runs on a PC. The program coded in Visual C# 2008 (Microsoft, Seattle, WA, USA) is responsible for centering the stage, initializing the system, and counting and locating the bacterial colonies. An electroluminescent screen (PES-A6W parallel electrode system, Knema LLC, Shreveport, LA, USA) is placed under the petri dish to provide back illumination. The top imaging camera is equipped with an imaging lens (*f* = 8 mm, M118FM08, Tamron, Saitama, Japan) with a viewing angle of 34 × 25.6° to cover the whole area of the plate within a given imaging distance (D_2_: 4.5 in.). The screen is turned on during the locating/counting phase to improve contrast. A schematic diagram of the proposed system is shown in Figure 1.

The forward scatterometer consists of a circular-beam laser module (Coherent Inc, Santa Clara, CA, USA), a 45 mirror, and a monochrome camera that is positioned just below the stage. The laser module has a wavelength of 635 nm and an output power of 0.95 mW. The camera used in the scatter module is the same as the colony imaging camera except for an added neutral-density filter (OD 2.5, Edmund Optics, Barrington, NJ) to prevent saturation of the CCD detector. The captured scatter patterns are imported to the colony-centering software, which performs automatic fine adjustment of the culture dish position to obtain circularly symmetric scatter signatures.

Motion control includes two stepper motors (PK243-01AA, Oriental Motor U.S.A. Corp, Torrance, CA, USA) driven by stepper-motor driver (MMD-17, Advanced Micro Systems, Inc, Essex Junction, VT, USA); the drivers are controlled by a four-axis motion-control chip (UMX-26, Pro-Dex Inc, Beaverton, OR), which produces a half-duty cycle square-wave step pulse to control the motors under various operation speeds. Two motors control the movement of the stage in the *R* direction and rotation of the petri dish in the *θ* direction, respectively. Both stepper motors have a step angle of 1.8°. For rotation, the motor is attached to a gear with 24 teeth and drives a larger gear below the petri dish having 192 teeth, providing a resolution of 0.225° per step. Equivalently, for a petri dish with a diameter of 88 mm, the linear resolution is about 180 microns at the edge of the dish. For linear movement of the stage, the motor is connected to a lead screw having a pitch of 0.25 in. per revolution, resulting in a resolution of 0.00125 in., or 31.75 μm per step. The UMX-26 motion controller is also connected to a PC via a USB 2.0 interface, and is controlled by passing ASCII command strings to the chip. The controller provides a range of velocities from 0 to 1.044 × 10^6^ step pulses per second and a range of accelerations from 0 to 8 × 10^6^ pulses per second, where 400 pulses constitute a single revolution. A universal power supply (Smart-UPS 1400, American Power Conversion, W. Kingston, RI, USA) with 1400 VA capacity was used as a system power source when a field test was performed.

### Software

**Figure 2:**
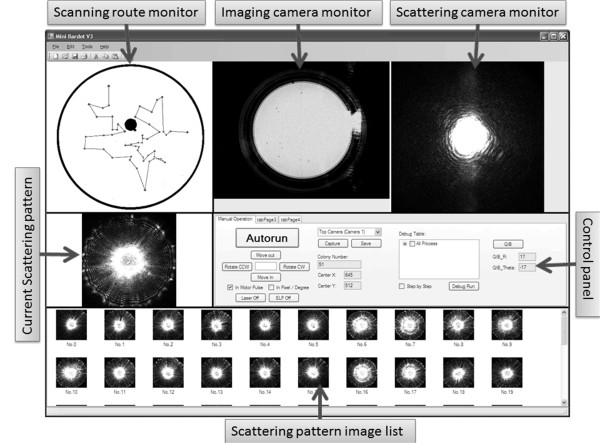
** Graphic User Interface.** The interface has two monitors for the imaging and scattering cameras, respectively; the control panel provides system configuration options and tools for manual control of the system. Other windows in the interface help the user to monitor the whole plate-scanning procedure.

As shown in Figure 2, a custom-built package was developed for automatic detection of scatter signatures. The software operates in of three phases: stage initialization, colony locating, and colony centering.

 1) Stage initialization In the first phase, the stage is centered with respect to the imaging camera for acquisition of the whole petri dish image. Since the radius of the dish is fixed, it is possible to pick the circular outline of the dish rim in the image as a reference object. Application of the Hough transform [20-22] enables calculation of the distance between the center of the image and the reference outline.

 2) Colony locating The colony counting and locating phase has five distinct steps: spatial filtering, colony separation, colony counting, colony location, and acquisition of morphometric characteristics. The spatial-filtering step resizes the image to 750 × 750 pixels, and assigns all HIGH (255) values to the area outside the petri dish. The colony-separation step binarizes the 8-bit image assigning 0 values to colonies and 1 values to the background. The binarization involves subtracting a pre-recorded background image from the actual plate image, and a segmentation process employing a region-growing algorithm to isolate the bacterial colonies [23,24]. The colony-locating step calculates the centroid of all the colonies and transforms Cartesian coordinates into polar coordinates. The final step calculates two morphometric parameters of the bacterial colonies: diameter and circularity. The diameter of a colony is expressed in pixels, and is computed using the following formula:

(1) $$D=2\sqrt{\left(\frac{A}{2\pi }\right)}$$

where *A* is the area of the colony in pixels, namely the total number of pixels found during colony segmentation. Meanwhile, the circularity is calculated as

(2) $$C=\frac{4\pi A}{p^{2}}$$

where *A* is the area of the colony in pixels and *p* is the perimeter of the colony in pixels . The measurement of the perimeter is calculated as [25]:

(3) $$\text{perimeter}=0.948\times {\text{n}}_{\mathrm{even}}+1.304\times {\text{n}}_{\mathrm{odd}}$$

where n_even_ is the number of even codes in the chain-code [26] representation of the colony edge and n_odd_ is the number of odd codes in the chain code. After locating the colony and obtaining the morphometric descriptors, the system performs a colony selection process, as colonies with diameters smaller than the predefined values or with low roundness values are excluded from the further scanning. Non-circular colonies are generally formed by the intersection of two or more separate colonies.

 3) Colony centering At the beginning of the colony-centering phase, optimum scanning trajectories through all selected colonies are computed by applying a generic algorithm to the equivalent traveling salesman problem. Bae et al. [18] showed that the generic algorithm provided efficient optimization results in a reasonable computation time . Following the given routine, the motion-control system moves the dish to the desired position, where the incident laser beam aims at the colony centroid location. However, since this centroid location is not identical to the centroid of the laser spot, macroscopic (coarse) adjustment is required to bring the two locations close to each other. Following this coarse movement, the software calculates the geometric centroid of the scatter pattern, which indicates the centeredness of the forward-scatter images. The system automatically performs fine adjustment to align the measured centroid with the center of the laser beam until either the difference is smaller than the threshold or the maximum number of iterations [25] is reached. In the colony-separation step, background subtraction and thresholding is critical to ensure sound colony locating. To perform this in automatic fashion, the threshold to separate the colonies is given by an empirical formula:

(4) $$\text{threshold}=40+\frac{1}{75}r+\frac{1}{6000}r^{2}$$

where *r* is the distance of a pixel from the center of the image.

### Elastic light scattering

ELS is defined as an optical measurement technique that utilizes the characteristics of the spatial distribution of the scattered light. ELS signal strength is very high compared to other spectroscopic and inelastic scattering techniques. By analyzing the ELS signal, it is possible to solve an inverse-scattering problem without any specific labeling reagent such as nucleic acid (DNA or RNA) or antibody probes, fluorophore molecules, or enzymes. The explanation of the ELS pattern differentiation originates from considering the bacterial colony as an optical amplitude/phase modulator [16,17]. When the photons from the laser interact with the micro structures of the colonies, the 2-D spatial distribution of the incoming wavefront is modified depending on the morphological differences of the individual colony. Therefore, upon leaving the far side of the colonies, the departing wavefront is now encoded with different amplitude and phase modulation in 2-D space which can be theoretically modeled via the Huygens-Fresnel principle in rectangular coordinate [16,17]. When this disturbed wavefront is propagated to the imaging plane, the characteristic scattering pattern is generated via spatial interference.

Compared to optical microcopy, ELS provides high throughput results and correlates well with the three dimensional structures. In addition, ELS does not require any types of label or stain to interrogate biophysical characteristics. Microscopy provides the real image of the object wheras ELS provides a transformed image that requires a solution of inverse problem to actually image an object. However, if the purpose is to discriminate among minute morphological changes, ELS identifies the sample in a fast, accurate, and label-free way.

### Pure and mixed plate experiments

Four different bacterial genera were scanned for single-species testing. Bacterial colonies were prepared as outlined in the material and methods section and three steps (stage initialization, colony locating, and colony centering) were performed automatically.

**Figure 3:**
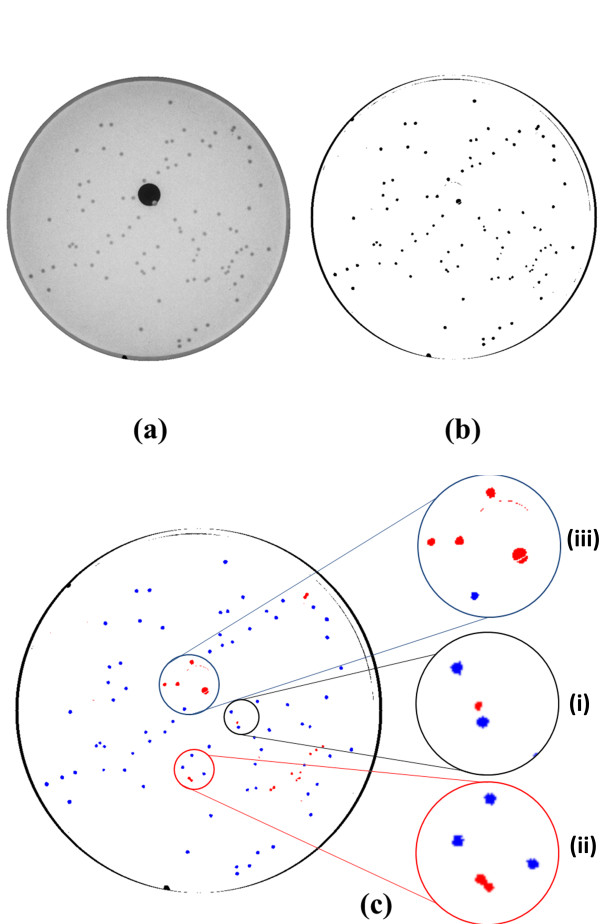
** (a) Spatial filtering and colony counting.** Raw plate image captured by the imaging camera. (**b**) Separation of colonies by subtracting the pre-saved background image and applying threshold. (**c**) Identification of colonies to be scanned by marking with blue color. (**i**) Colonies with too small or large diameters; (**ii**) colonies with low roundness values (indicating two or more intersecting colonies); (**iii**) colonies too close to the black hole in the raw plate image.

**Figure 4:**
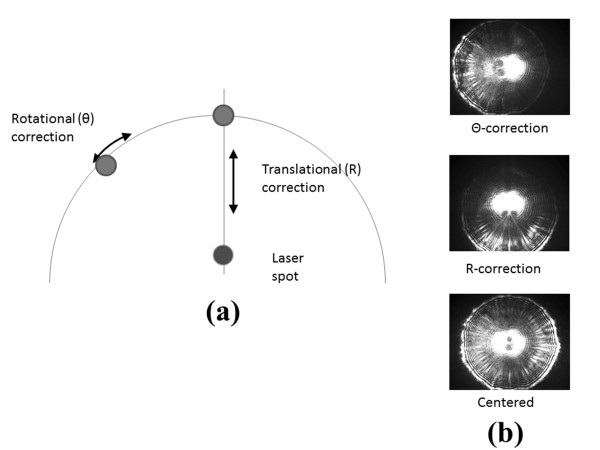
**Centering mechanisms for R-θ coordinates.** (**a**) The first step is to rotate the colony to fall under the center line and then to move the translational stage to the laser spot. (**b**) Actual scatter patterns with centering steps for *Listeria innocua* F4244.

**Figure 5:**
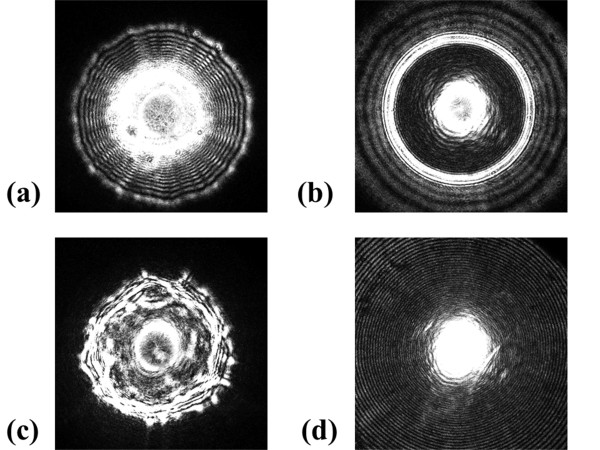
** Representative scatter images for four different genera.** (**a**) *L. innocua* F4244, (**b**) *E. faecium,* (**c**) *E. coli,* and (**d**) *S. aureus*.

Figure 3 shows the result of colony-counting steps. After the raw image is captured from the camera (Figure 3(a)), background is subtracted before segmentation. Then, the system automatically marks the colonies that satisfy the selection criteria. These colonies are queued into the trajectory optimization algorithm (Figure 3(c)). Colonies whose position lie close to the incident laser passage, or whose diameter is too small, or whose lack of circularity indicates a union of two colonies are eliminated from the scanning sequence. Finally, Figure 4 shows the centering mechanism in R-θ coordinates. Once the distance between colony centroid and laser center is known, rotational correction is performed to align the colony with the laser. Then a linear correction is performed to reduce the difference between the two centers to less than a pre-defined threshold. These coarse correction steps are followed by fine correction steps to provide circularly symmetric scatter patterns; an average of 6.67 fine adjustment steps per colony was required for 480 tested colonies. The typical average measurement speed for all three steps is approximately 2.7 colonies per minute; representative final scatter images are shown in Figure 5 for *L .innocua* F4248, *E. coli* stbl, *E. faecium,* and *S. aureus*.

**Figure 6:**
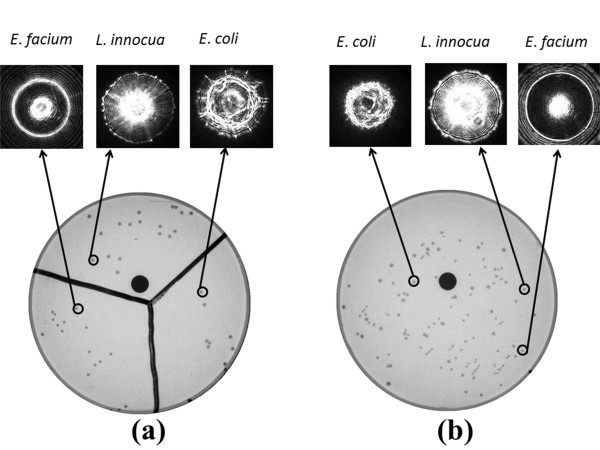
** Petri-dish image of mixed plate experiment.** (**a**) bacteria inoculated separately in different regions; (**b**) completely random inoculation.

Mixed plate experiments were then conducted. Among the four genera, three types of bacteria were selected; *L. innocua* F4248, *E. coli* stbl, and *E. faecium.* Mixed-plate experiments were performed in two steps. First we divided a single petri-dish in to three equal regions and inoculated the known bacterial genera. Figure 6(a) displays the three regions with different types of bacteria. Then we mixed all three genera and inoculated simultaneously without designating any boundary or regions. Nevertheless, the three genera were clearly separated by their distinctive ELS patterns (Figure 6(b)). Even though differentiation at genera level was shown, the discriminatory power of ELS has been proved to characterize at species level and in some cases, at strain level [27]. This was possible through combing the sensitivity of the ELS and quantitative classification software using Zernike and Haralick features

### Comparison with forward scatterometer

**Figure 7:**
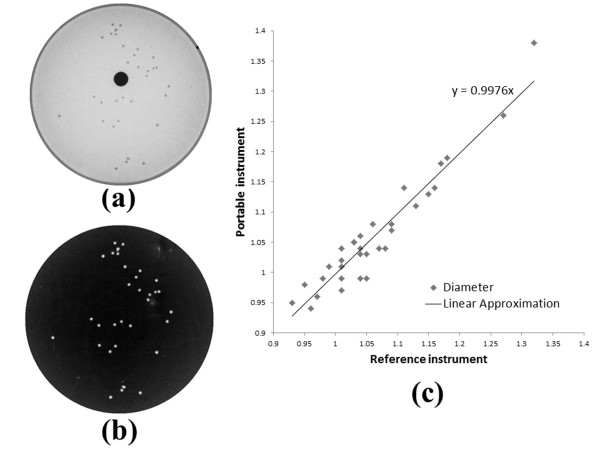
** Diameter comparison.** The same plate of *L. innocua* F4244 was scanned in both the portable and the reference forward scatterometer to correlate the captured diameter of individual colonies.

Since the design aim of the portable instrument was operation as a field-deployable unit, we have compared the scatter patterns captured by the portable instrument and by the reference forward scatterometer located in the laboratory. Figure 7 shows the plate image from (a) the portable and (b) the reference instrument; the proposed system displays a black circle in the center area where the laser passes through. An important parameter that allows correlation of measurement between the two instruments is the colony diameter estimation, as it is used to select colonies for further analysis. Therefore, we compared the estimated diameters calculated from the images captured from both instruments and plotted the diameter correlation, as shown in Figure 7(c) (least square with R^2^ = 0.9191). Most diameter values were close to each other; the error for the remainder was within 3-7%. In Figure 7, the maximum difference in percentage is 7.22%, and most pairs resulted in a difference of less than 3%. Since the image size of the 88-mm plate is 750 × 750 pixels, one pixel measures approximately 0.12 mm of physical length. Therefore, the typical diameter of a bacterial colony was 1-1.2 mm, or 10 pixels in one dimension; depending on the local noise level, a single-pixel difference in diameter measurement can cause the percentage error observed in the differences.

### Field testing

**Figure 8:**
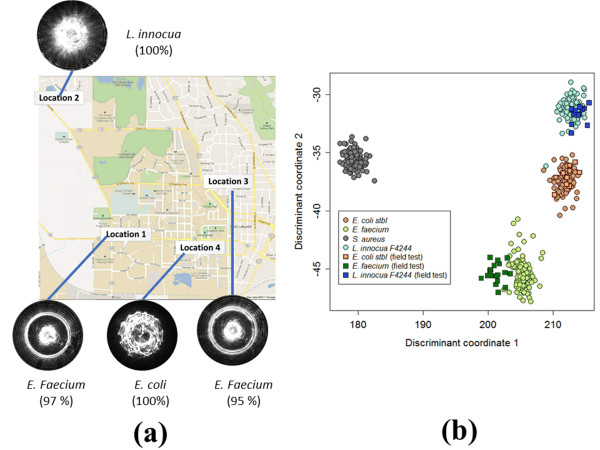
** Field-test results with representative scatter patterns and classification accuracy.** (**a**) The portable scatterometer was installed in a vehicle and operated via an UPS; only three different organisms were tested owing to safety regulation. The scans were performed in four different locations with their GPS position recorded and the reference database was accessed using a commercial cellular network. (**b**) Comparison between the reference and portable scatterometer.. Circles denote the features extracted from database constructed with reference scatterometer system (red-*E.coli*, blue-*L. innocua,* green-*E.facium*, and gray-*S.aureus*) while squares represents the features from the field test of the portable detector.

Assuming a security threat from bioterroism, field-deployable units can play an essential role in the process of identifying of the unknown agent. Since this device can be built for lower cost than reference instruments, it can be deployed in multiple locations to monitor and report any suspicious activity via ELS signatures of both clinical and food-borne bacteria. To emulate this scenario and provide a feasibility study of field-deployability, we operated the described instrument in a vehicle driven to four different locations near the Purdue University campus. Owing to safety regulations, only three genera (*L .innocua* F4248, *E. coli* stbl, and *E. faecium*) were prepared in the laboratory for the field testing. The representative scatter image and a list of measured species are shown in Figure 8(a). Table 1 provides the GPS location (ThinkVantage GPS, Lenovo), the bacteria tested, and the final classification accuracy. Once the forward-scatter patterns were captured, the automated feature extraction following the process described previously was performed. The extracted features were used by a remotely operated SVM classifier to automatically recognize the type of bacteria forming a particular colony [28,29]. The pattern database and the classifier were accessed through a commercial cellular network (AT&T HSDPA 3.6 Mbps); the overall classification accuracy for the three tested species was above 95%. Figure 8(b) shows the discriminant coordinates plot of randomly selected training colonies (characterized using the reference forward scatterometer), and projected scatter-patterns that were collected during the field test. Discriminant coordinates 1 and 2 are defined as a linear combination of the Zernike and Haralick features that maximizes the distance between instances in different groups and minimizes the distance within groups. Although the actual classifier employed in our system is SVM-based, the 2-D plot of linear discriminate coordinates illustrates well the reproducibility and compatibility between the lab system, and the portable instrument. Circles represent the colonies characterized by scatter patterns measured with the regular lab system, while the squares represent data acquired using the portable device.

**Table 1:** Field-test data: GPS location, types of bacteria, and their classification accuracy

	**GPS Coordinate**	**Bacterial species**	**No. of colonies**	**Mean accuracy**
Location 1	Lat:40.422287 Long:-86.927704	*E. faecium*	21	97%
Location 2	Lat:40.44557 Long:-86.942497	*L. innocua*	25	100%
Location 3	Lat:40.430313 Long:-86.910759	*E. faecium*	18	95%
Location 4	Lat:40.420986 Long:-86.916092	*E. coli*	11	100%

The great advantage of the proposed instrument is shown by the field-testing results. Even though the experiment was performed locally around the campus, we can easily imagine the impact of using the portable device for resource-scarce areas. The screening result can be delivered wirelessly to the local instrument by accessing thousands of reference ELS images using feature extraction. The only drawback of the proposed system is that the system requires the bacterial colony to be grown on an agar medium. However, compared to the other reagents and expensive instruments needed for other types of screening, the petri-dishes and media can be considered as cost-effective consumables. In addition, numerous rapid methods require the plating of the bacteria anyway for final confirmation of the identification.

For hardware designs, since the UPS can provide 1400 VA of power and the consumption rate of the overall system is high, a single UPS can sustain about 15 min of continuous operation for the portable scatterometer and its accessories. This means that we can scan two plates of bacterial samples with an average number of 15-20 colonies per plate. In the future, both a higher-capacity power source and greater energy efficiency will be incorporated into the overall design of the system to maximize the measurement capability along with a modified optics to reduce the number of CCD cameras. Finally, the laser pointing stability is a critical issue when centering between the incoming laser and the colony center; measurements performed in a moving vehicle resulted in an increased number of centering steps or in poor scatter images. Therefore, all scanning and image acquisition were performed when the vehicle was parked.

## Conclusions

A field-deployable bacterial identification system that uses elastic light scatter images has been introduced. The proposed device can provide more rapid identification of the source of an outbreak for resource-poor areas via wireless access capability to a central database, which can play an important role in constructing a national/international surveillance network. The portable device weighs 9 lb, is 12 × 6 × 10.5 inches, and is designed to access a scatter-fingerprint database using a commercial cellular wireless network. The hardware design includes a linear and rotational mechanism to provide a small footprint and incorporates various algorithms for colony locating and centering. Four representative bacterial genera, *L. innocua* F4248, *E. coli* stbl, *E. faecium,* and *S. aureus,* were used for single-species testing and comparison with the reference forward scatterometer for database compatibility. Finally, a field test was conducted at four different locations and resulted in classification rates of 95-100%.

## Methods

### Sample preparation

Four different bacterial genera were selected as representative samples: *Listeria innocua* F4248, *Escherichia coli* stbl, *Enterococcus faecium,* and *Staphylococcus aureus*. Trypticase soy agar (BD, catalog #211043) was used for sample preparation. 40 mg agar powder was suspended in 1 L Millipore water, boiled for 1 min, and allowed to cool. Twenty-five ml cooled agar was dispensed onto sterile 88-mm round petri dishes. Samples were serially diluted and 25 μl bacterial cultures was dispensed into the center of each petri dish. An L-shaped spreader (RPI Corp. #247660) was used to spread the bacterial dilution on the agar; the plate was then placed in a 37°C incubator.

## Competing interests

The authors declare that they have no competing interests.

## Authors’ contributions

EB, DY, and DK contributed to the design of the hardware and programmed the operating software. VP and BR had developed and analyzed the SVM classification software; CH and JS provided the microbial samples for testing; VJD and JPR provided the guidelines for the overall project and contributed to the wireless database. All authors read and approved the final manuscript.
